# Effect of in-situ aged and fresh biochar on soil hydraulic conditions and microbial C use under drought conditions

**DOI:** 10.1038/s41598-018-25039-x

**Published:** 2018-05-01

**Authors:** Lydia Paetsch, Carsten W. Mueller, Ingrid Kögel-Knabner, Margit von Lützow, Cyril Girardin, Cornelia Rumpel

**Affiliations:** 1 Chair of Soil Science, Technical University of Munich, Emil-Ramann-Strasse 2, 85354 Freising, Germany; 2 Institute for Advanced Study, Technical University of Munich, 85748 Garching, Germany; 3 INRA, ECOSYS, UMR INRA-AgroParisTech, Thiverval-Grignon, 78850 France; 4 CNRS, IEES, UMR (Sorbonne Université, UPEC, IRD, INRA, CNRS), Thiverval-Grignon, 78850 France

## Abstract

Biochar (BC) amendments may be suitable to increase the ecosystems resistance to drought due to their positive effects on soil water retention and availability. We investigated the effect of BC *in situ* ageing on water availability and microbial parameters of a grassland soil. We used soil containing ^13^C labeled BC and determined its water holding capacity, microbial biomass and activity during a 3 months incubation under optimum and drought conditions. Our incubation experiment comprised three treatments: soil without BC (Control), soil containing aged BC (BC_aged_) and soil containing fresh BC (BC_fresh_), under optimum soil water (pF 1.8) and drought conditions (pF 3.5). Under optimum water as well as drought conditions, soils containing BC showed higher soil organic carbon (SOC) mineralization as compared to control soil. Moreover, BC effects on the soil water regime increase upon *in situ* aging. Native SOC mineralization increased most for soils containing BC_aged_. The BC_aged_ led to improved C use under drought as compared to the other treatments. We conclude that BC addition to soils can ameliorate their water regime, especially under drought conditions. This beneficial effect of BC increases upon its aging, which also improved native substrate availability.

## Introduction

A major challenge of climate change is the increasing frequency of extreme hydrological events such as droughts (IPCC 2007), which will have strong impacts on terrestrial ecosystems and the biogeochemical carbon (C) cycle^1^. In particular, droughts can affect the quantity and quality of organic matter (OM) retained in soil, as soil moisture is one of the most important factors driving microbial processes. Recently, addition of biochar (BC) to soil was suggested to ameliorate water retention under drought conditions^2^. Biochar is a highly aromatic material produced by thermal degradation of organic materials with limited or no air supply and is distinguished from charcoal by its use as a soil amendment^3^. Its addition to soil was found to alter the soil’s physical structure and air capacity^4^ and to be beneficial for water holding capacity (WHC), water retention, and plant available water^5,6^. Biochar (BC) is proposed as beneficial for soil improvement but the usage competition of organic material as feedstock and economical aspects are mayor points of criticism for agricultural scale usage as soil amendment^7^. Moreover, the aging of BC and the interpretation of its effects on soil physical and microbial parameters suffer from a strongly varying composition and characteristics of the BC. To overcome these common critical issues, we used a highly homogeneous BC, produced at industrial scale – a waste product from heat production.

In mineral soils, drought reduces the microbial activity^8^ due to physiological stress and limited substrate supply to microbial cells^9^. Microorganisms can react to this physiological stress by physiological changes or by a shift of their communities towards microbes with higher water stress resistance^10^. Highly stressed microbes will then use substrate for maintenance and not for growth^11^, which ultimately affects their metabolic efficiency.

Microbial activity response to drought was found to be dependent on soil organic matter quality^12^ and soil properties^13^. Biochar addition, depending on BC feedstock^14^ and production conditions^15–17^, may strongly affect hydraulic properties^18^ and thereby alter microbial activity under drought conditions.

The degradation of BC is assumed to be mostly microbial induced, but Zimmerman^19^ and Cheng, *et al*.^20^ showed that abiotic BC-C release due to processes such as chemical oxidation, photooxidation, or solubilisation can significantly contribute to the C release.  Aging after field exposure can significantly alter physico-chemical parameters of the BC^21^ and therefore most probably its effects on soil parameters^22,23^. Biochar addition to soil may also change microbial community composition^24^. An indicator used to determine the microbial use of carbon is the metabolic quotient (qCO_2_), considering the respiration rate CO_2_-C per unit microbial biomass C^25^. The qCO_2_ was found to decrease by 13% after BC amendment compared to the control indicating improved soil biophysical conditions^26^. While the microbial biomass responded with growth, the total soil CO_2_ production remained unchanged after BC amendment.

Biochar properties strongly changed as a consequence of short term field exposure of less than 6 months. In particular, increasing surface area and changes in chemical properties were observed^27–29^, recently discussed as caused by organic coatings^30,31^. This resulted in increasing wettability of the BC over time, and hence, a better availability for microbial degradation^32^. Artificial weathering induced through chemical and/or physical treatments increased carbonyl and carboxylic functional groups as well as the biological stability of the residual BC^33,34^.

We investigated the effects of physico-chemical changes occurring in BC amended soils with time of field exposure on the response of microbial parameters to drought stress. We hypothesized that BC aging impacts soil hydraulic properties and affects microbial activity under drought conditions. We set up an incubation experiment with soil containing ^13^C labeled BC (1) added to soil after production (BC_fresh_) and (2) sampled after 3 years of field exposure (BC_aged_). The ^13^C labeling allowed us to monitor mineralization of native SOC in addition to total SOC. Our specific objectives were to quantify the effect of fresh and aged BC (i) on soil hydraulic properties ii) microbial biomass, its activity and metabolic quotient and iii) native SOC mineralization. The soils were incubated under two different water potentials to investigate the impact of drought on these parameters.

## Material and Methods

### Study site

The soil used in this study was sampled from a BC field experiment located at the site of the long-term field experiment “SOERE ACBB” managed by INRA (National Institute of Agricultural Research) in Lusignan (46°25″12.91″N; 0°07″29.35″ E), France. Mean annual rainfall is 800 mm and the mean annual temperature is 11 °C. The soil is classified as a Dystric Cambisol^35^ with loamy texture (11% sand, 72% silt and 17% clay), a bulk density of 1.4 g cm^−3^ and a pH of 6.0 (control soils) and 6.4 (biochar plots). It was considered that total C concentrations represent organic carbon (OC) because we did not find any evidence of inorganic C contribution to our soils using acid treatment. The OC and total N content was 1.4% and 0.16%, respectively^36^. Before 2012, the field was a temporary grassland (C_3_ plants) and is now cultivated with *Festuca arundinacea* and *Dactylis glomerata*. The field experiment comprised 8 plots of a size of 16 m²: 4 BC amended plots and 4 control plots. A single addition of 3 kg m^−2 13^C labeled BC to the upper 10 cm took place in May 2012 by a rotary hoeing. After three years, BC amendment led to pH increase from 6.0 (control) to 6.4 (BC). An aliquot of the BC used for the field experiment was stored in the laboratory for three years for reasons of comparison.

### Biochar production and parameters

The BC was produced by gasification of maize (*Zea mays* L.) (C_4_ plant) silage (~10 mm) for 40 min at 1200 °C (heating rate 26–40 °C/min) in a commercial reactor (©A.G.T. – Advanced Gasification Technology s.r.l., Cremona, Italy). The shape and size of the pellets did not change during gasification. General parameters of the gasification BC used for the experiment are listed in Table 1.

**Table 1:** General parameters of the biochar.

**Parameter**	**Unit**	
pH (0.01 *M* CaCl_2_, 1:2.5 w-vol)		10.1 ± 0.2
Salinity	mS cm^−1^	9.6
Ash (550 °C)	% DM	10.3 ± 1.7
Carbon	%	69.5 ± 1.3
Nitrogen	%	1.6 ± 0.1
C/N		41
H/C		0.4
O/C		2.9
δ^13^C	‰	−13.7 ± 0.1

### Sampling and Pre-treatment

For the incubation experiment disturbed soil samples were randomly taken from the top 10 cm from the eight soil plots in September 2015. Disturbed samples were stored in plastic bags and transported to the laboratory. We did not sieve the soils to retain BC pellets >2 mm. Plant residues and roots were manually removed. In addition, we collected three undisturbed (100 cm³ sampling rings) soil samples from each of the eight plots for determining soil water retention curves of control soil and BC amended soils.

### Incubation Experimental Design

**Figure 1:**
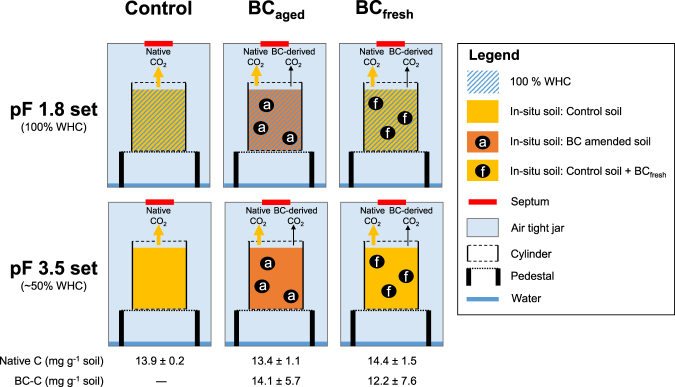
Experimental set-up: Each amendment and set had three replicates, analyzed at five time points (n = 30). Control soil was soil from the field experiment without BC addition. BC_aged_ was disturbed BC amended soil of the field experiment 3 years after field exposure. BC_fresh_ was control soil from the field experiment mixed with fresh BC.

The experimental design is presented in Fig. 1. Incubations were carried out in triplicates with three different treatments: control soil from the field experiment, BC containing soil from the field experiment sampled three years after the amendment (BC_aged_) and control soil from the field experiment amended with fresh BC (BC_fresh_). BC_fresh_ was the same BC as used for the field experiment but stored dry and dark for three years in the laboratory. The BC amounts added were chosen in accordance with the amount of BC per amount soil C in the field, which had been determined before using the C content and δ^13^C ratio of BC amended soils from the field experiment.

We filled 160 g of the sample into plasic cylinders and compressed it to a bulk density of 1.4 g cm^−^³ (according to the *in-situ* bulk density). Afterwards, these samples were drained in pressure plate extractors at pF 1.8 and 3.5 to set up the experimental water conditions, representing a water holding capacity (WHC) of 100% and about 50%, respectivly.

The cylinders were then placed in 1 L glass jars and 10 ml distilled water were added to the jars to maintain soil humidity. In total the experiment consisted of 90 jars. All jars were flushed for 20 min with moistened CO_2_ free-air to remove CO_2_ from the jars atmosphere and capped with air-tight lids. The jars were incubated in the dark at a constant temperature of 20 °C to minimize abiotic degradation by photooxidation. Decomposition was measured by monitoring the CO_2_ release at days 2, 7, 15, 28 and 90 using a MICROGC (Agilent, Santa Clara, USA). Therefore gas was sampled in the headspace with a syringe and injected in a GC analyser to determine CO_2_ concentration. Another gas sample was injected into the GC/IRMS system (isotopic ratio mass spectrometer; Microgas) to measure the ^13^C isotope signature of the CO_2_. This allowed to distinguish C_4_-BC mineralisation from native C_3_-SOC mineralisation using the isotopic mass balance. After gas measurement, the jars were flushed and sealed as discribed above. At the measuring days whole sample sets were removed to determine water content, microbial biomass and contents of C and N. To avoid anaerobic conditions, we monitored the CO_2_ and flushed the jars at least every 14 days with synthetic air throughout the incubation period.

The C and N concentrations of bulk soils and fresh BC were measured by dry combustion with an elemental CNS analyser (elementar vario MAX CUBE, Hanau, Germany). The δ^13^C signature of the pure fresh BC was δ^13^C = −13.7‰ and of the C_3_-SOM δ^13^C = −27.3‰. As reported before^37^, there was no change in the δ^13^C from fresh to weathered BC.

### Microbial biomass

Extraction of microbial biomass was carried out after modification of the chloroform fumigation extraction method^38^. Each sample was divided into two sub-samples of 10 g: a non-fumigated reference sample and a sample fumigated with chloroform. The fumigated samples were incubated under ethanol-free chloroform (CHCl_3_) vapor in a desiccator for 16 h, followed by 6 vacuum-purge cycles to remove the CHCl_3_. Both sets were extracted with 40 ml of 0.03 *M* K_2_SO_4_, shaken in an overhead shaker for 30 min and centrifuged for 10 min with 10,000 RPM. The supernatants were removed, filtered, frozen to −20 °C and freeze dried. Organic C and N content as well as δ^13^C signature of the K_2_SO_4_-extractable C were analyzed using an isotope ratio mass spectrometer (Delta V Advantage Thermo Fisher Scientific, Bremen, Germany) coupled with an elemental analyzer (Flash 2000, Thermo Fisher Scientific, Bremen, Germany). All δ^13^C values were expressed relative to the Pee Dee Belemnite (PDB) international isotope standard.

### Calculations and statistics

The proportion of native C in the microbial biomass (*bC*_3_) was calculated by the two component stable isotopic mixing model approach (IMM) after Balesdent and Balabane^39^:

1 $$b{C}_{3}=\frac{\delta {C}_{mixture}-\,\delta {C}_{4}\,}{\delta {C}_{3}-\delta {C}_{4}}$$

where δC_4_ is the δ^13^C isotope signature of the pure BC and δC_3_ of the native SOC of the control soil. δC_mixture_ is the δ^13^C signal of the soil sample extracts. The same model was used to partition the BC-C contribution to the CO_2_ efflux. For the determination of respired BC-C, the BC-C proportion were multiplied by accumulated CO_2_–C. Correspondingly, remaining BC-C in the soil was calculated by subtracting mineralized BC-C from total BC-C added at the beginning of the experiment. The extent of the priming effect of the biochars on native SOC mineralization were calculated as the difference between the CO_2_ efflux from SOC in the control compared to the native CO_2_ efflux of native SOC from soil-biochar mixtures, estimated with the two-component isotopic mixing model.

Microbial biomass was calculated by dividing the measured OC concentrations per g dry soil by the factor k_EC_ = 0.45. The extractable C of the non-fumigated set were used as approximated values for salt-extractable organic carbon (DOC).

The microbial biomass based metabolic quotient (qCO_2_) was calculated to evaluate the microbial C use efficiency and the substrate availability. A high metabolic quotient may indicate a low efficiency of C mineralization and a higher substrate availability^40^. The qCO_2_ was calculated by using the equation

2 $$qC{O}_{2}=(\frac{C{O}_{2} \mbox{-} C}{{C}_{mic}})$$

where CO_2_-C is the cumulative respired CO_2_-C in mg kg^−1^ soil h^−1^ and C_mic_ the corresponding microbial biomass C in g kg^−1^ soil.

By using the software SHIPFIT2.0^41^, we fitted the water retention characteristics to the data by using the unimodal Kosugi retention function^42^ given by:

3 $${\Gamma }(h)=\frac{1}{2}\,erfc\,{[\frac{ln(\frac{h}{{h}_{m}})}{\sqrt{2}\sigma }]}^{}$$

where *h*_m_ (L) is the pressure head corresponding to the median pore radius, *σ* (–) is the standard deviation of the log-transformed pore-size distribution density function, and erfc() is the complementary error function. For unsaturated soils *h* (L) is defined as positive.

For statistical analyses we used the software RStudio, version 3.3.1 for Windows^43^. Significant differences between the amendments and control were tested with a one-way analysis of variance (ANOVA).

## Results

**Figure 2:**
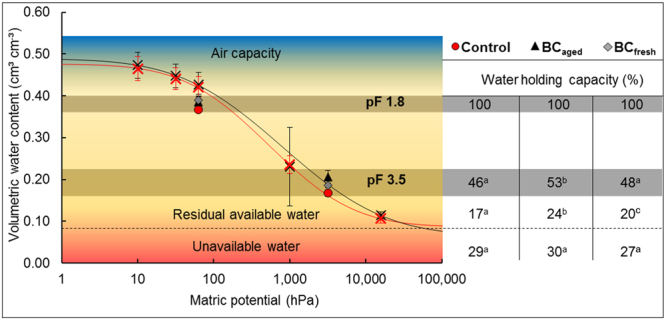
Volumetric water contents of the undisturbed soil samples (red (control) and the black crosses (BC_aged_) (n = 12) fitted with the model of Kosugi^42^ (lines)). The water holding capacities (WHC) of the pF 1.8 and 3.5 sets (n = 30) were determined after the incubation. Data within one row with different letters are significantly different (p < 0.05).

While the water retention curves of undisturbed field samples showed little differences for plots with and without BC, in the incubation experiment, volumetric water contents of BC containing soils at pF 3.5 were significantly increased compared to the control (Fig. 2).

Moreover, after incubation, BC_aged_ showed higher volumetric water contents compared to BC_fresh_ at pF 3.5. Air capacity as well as plant unavailable water (>pF 4.2) was not affected by the amendments. However, the presence of BC_aged_ increased remaining plant available water under drought conditions (pF 3.5 to 4.2) compared to control soil and soils containing BC_fresh_.

Carbon and N contents are given in Table 2. Following BC addition (aged or fresh), the SOC contents doubled, whereas SON contents increased only by about 16%. Consequently, C to N ratio increased from 9.8 for control soil to 16.5 and 15.7 for soils containing BC_aged_ and BC_fresh_, respectively.

**Table 2:** Mean C contents, N contents and C to N ratios of bulk soils (n = 18) from 0 days to 90 days of incubation with standard deviations.

	pF value	C content	N content	C to N ratio
		mg g^−1^	mg g^−1^	
Control	1.8	13.88^a^ ± 0.41	1.44^a^ ± 0.03	9.62^a^ ± 0.17
	3.5	14.01^a^ ± 0.33	1.41^a^ ± 0.03	9.91^a^ ± 0.03
BC_aged_	1.8	28.27^b^ ± 4.36	1.65^b^ ± 0.08	16.83^b^ ± 1.54
	3.5	26.87^b^ ± 2.29	1.65^b^ ± 0.04	16.09^b^ ± 0.92
BC_fresh_	1.8	27.52^b^ ± 5.82	1.69^b^ ± 0.09	15.90^b^ ± 0.84
	3.5	26.38^b^ ± 3.21	1.69^b^ ± 0.06	15.55^b^ ± 0.59

Different letters within one column mark significant different values (p < 0.05).

**Figure 3:**
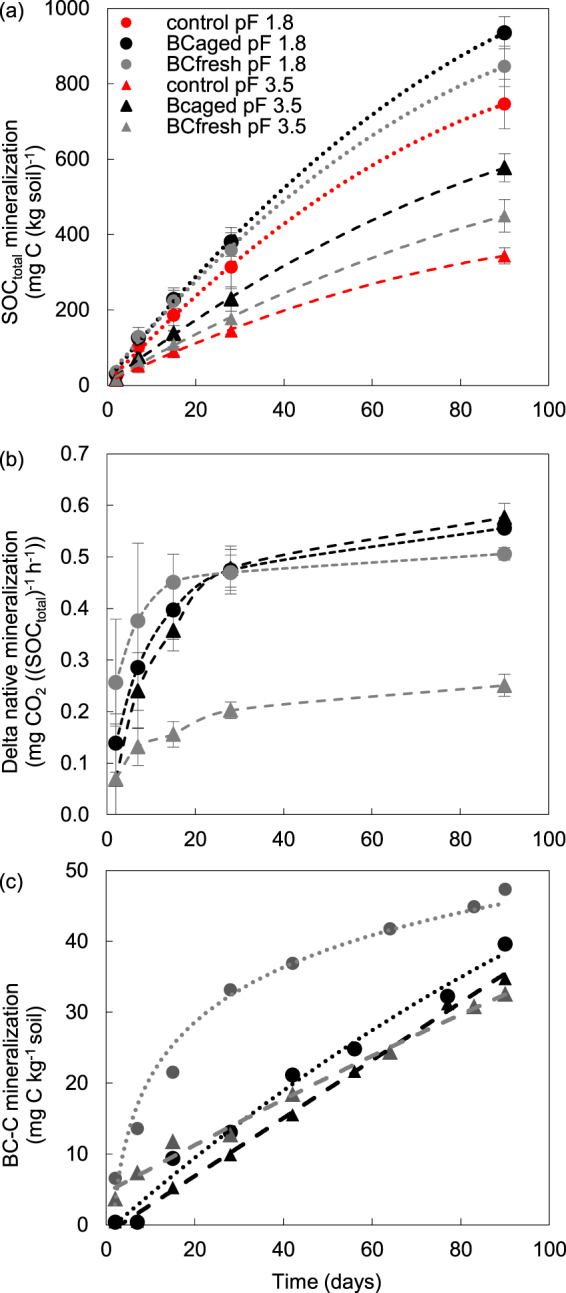
(**a**–**c**) (**a**) Cumulative CO_2_ emissions, (**b**) additional native mineralization (mg per kg SOC) in BC amended soil and (**c**) cumulative BC-C mineralization (mg per kg) during 90 days of the experiment. The vertical bars represent the standard deviation (n = 12).

The cumulative total C mineralization, native SOC and BC-C emissions during the 90 days of incubation are presented in Fig. 3a–c.

Our results show two separated groups with regard to the OC mineralization according to the soil water potential (Fig. 3a). The total respired C at pF 1.8 was higher than at pF 3.5 for all three treatments. Under optimum water conditions (pF 1.8), the highest C mineralization was observed for BC_aged_ (935.5 mg C kg^−1^ soil) followed by BC_fresh_ (846.5 mg C kg^−1^ soil) and control (734.0 mg C kg^−1^ soil). Under drought conditions (pF 3.5), these values decreased by 54% for the control soil and by 38% and 47% and BC amended soils (BC_aged_ and BC_fresh_, respectively).

Both amendments increased native SOC mineralization (Fig. 3b). The highest native SOC losses were found at optimum water conditions for BC_aged_ (896.3 mg C g^−1^ soil). This corresponds to a positive priming effect leading to 21% increased native SOC mineralization with regards to the control. The addition of BC_fresh_ induced a lower priming effect corresponding to 8% increase of native SOC mineralization as compared to the control. Under drought conditions, the increase of native SOC mineralization was even higher, with BC_aged_ leading to 59% and BC_fresh_ to 22% more native C loss compared to the control.

The BC-C contributions to the respired CO_2_-C were higher under drought than under optimum conditions (Fig. 3c). Biochar derived C in BC_aged_ soils accounted for 3.5% (pF 1.8) and 5.9% (pF 3.5) and in BC_fresh_ soils for 5.6% (pF 1.8) and 8.8% (pF 3.5) of the total mineralized C, over the 90 days of incubation period. However, low amounts of BC-C were mineralized during the 90 days of incubation. Less than 0.5% of BC-C was respired. Higher BC-C proportions were respired in soils containing BC_fresh_ (0.3% of BC-C) than in soils containing BC_aged_ (0.2% of BC-C). Despite a ‘BC-C flush’ at pF 1.8 after BC_fresh_ addition at the beginning of the experiment (Fig. 3c), the mineralization of both BC types differed only marginal after 90 days.

Under optimum water conditions, microbial biomass ranged between 155.4 ± 12.4 mg kg^−1^ soil to 171.2 ± 13.7 mg kg^−1^ soil at the beginning of the experiment. After 90 days of incubation, microbial biomass decreased in all treatments and ranged between 98.2 ± 6.2 mg kg^−1^ soil and 121.7 ± 3.3 mg kg^−1^ soil. Similar values were recorded for drought conditions (Table 3).

**Table 3:** Microbial biomass C (MBC) of all treatments during the 90 days of incubation. Asterisks indicate significant differences (p < 0.05).

Set		Unit	Treatment	Incubation time (days)				
				2	7	15	28	90
**pF 1.8**	MBC	mg C (kg soil)^−1^	Control	158*	142,9 ± 7.9	149 ± 4.2	108,4 ± 4.0	98,2 ± 6.2
			BC_aged_	171,2 ± 13.7	162 ± 1.0	154,7 ± 33.8	168,7 ± 34.0	111,2 ± 11.4
			BC_fresh_	155,4 ± 12.4	156,5 ± 16.6	160,9 ± 4.3	155,1 ± 3.7	121,7 ± 3.3
	Proportion BC-C	%	BC_aged_	−8,1 ± 0.4*	−7,1 ± 1.8*	−0,4 ± 1.4	−1 ± 1.0*	−1,4 ± 1.6*
			BC_fresh_	−4,6 ± 0.6*	−2,3 ± 1.5*	−0,7 ± 0.4	2,9 ± 1.0*	3,4 ± 1.7*
**pF 3.5**	MBC	mg C (kg soil)^−1^	Control	130,3 ± 9.2	138,6 ± 3.1	137,2 ± 12.3	136,1 ± 16.4	118,3 ± 13.9
			BC_aged_	137 ± 9.1	157,7 ± 37.4	145,7 ± 16.8	157 ± 42.1	132,8 ± 1.5
			BC_fresh_	151,7 ± 0.8	162,6 ± 5.5	153,1 ± 5.8	156,7 ± 4.2	162,2 ± 18.7
	Proportion BC-C	%	BC_aged_	−4,4 ± 1.2*	−5,8 ± 8.9	−0,8 ± 1.8	−2 ± 1.7	−4,3 ± 1.1
			BC_fresh_	1,2 ± 2.7*	0,2 ± 0.9	0,5 ± 1.4	2,5 ± 0.7	−1,2 ± 2.5
**pF 1.8**	MBC	mg C (kg SOC)^−1^	Control	10,9*	10,4 ± 0.3	11 ± 0.4	7,5 ± 0.4	7,3 ± 0.1
			BC_aged_	5,7 ± 2.2	7 ± 3.3	6,4 ± 1.4	6,7 ± 1.4	3,6 ± 1.2
			BC_fresh_	6,2 ± 3.7	4,4 ± 1.3	6,2 ± 1.1	7,4 ± 2.4	5,3 ± 1.5
**pF 3.5**		mg C (kg SOC)^−1^	Control	9,2 ± 0.6	9,9 ± 0.2	10,1 ± 0.5	9,5 ± 1.0	8,5 ± 0.6
			BC_aged_	4,8 ± 1.6	6,4 ± 3.5	5,5 ± 1.0	6,3 ± 3.1	5,3 ± 1.2
			BC_fresh_	4,6 ± 1.1	6 ± 0.9	5,8 ± 0.1	7,5 ± 0.7	5,9 ± 1.2

^*^No replicates.

Between the start and the end of the experiment, the microbial biomass of BC amended soils decreased by 35% and 38% at pF 1.8 (aged and fresh), whereas it remained rather constant at pF 3.5. The δ^13^C abundance in the fumigation extracts from control, BC_aged_ and BC_fresh_ were very similar resulting in high uncertainties for the calculation of the BC-C proportion (Table 3).

Within the first week of the incubation experiment, microbial biomass from all samples showed a depletion of ^13^C to differing degrees (up to 8.1 ± 0.4% for BC_aged_ at pF 1.8). This effect leveled out with progressing time but microbial biomass in the treatments BC_aged_ remained depleted in ^13^C at both water potentials compared to the control after 90 days of incubation. BC_fresh_ showed 3.4 ± 1.7% incorporation of BC derived C into microbial biomass under optimum conditions, whereas under drought, BC-C was not incorporated into the soil microbial biomass.

**Figure 4:**
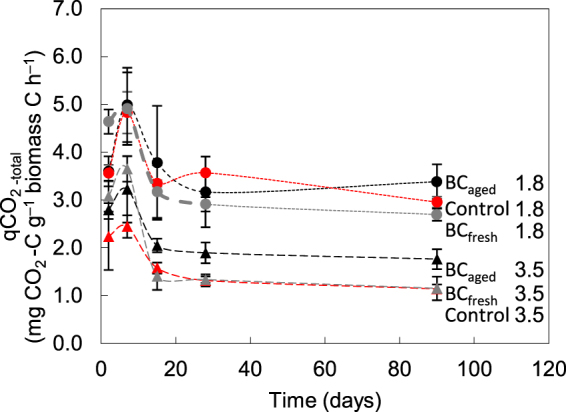
Metabolic quotient (qCO_2_) during the incubation experiment. Every data point represents a mean value of three replicates with standard deviation.

The metabolic quotient (qCO_2_) of the control soils decreased during incubation from 3.56 to 2.95 mg CO_2_ -C g^−1^ biomass C h^−1^ under optimum water conditions and from 2.24 to 1.14 mg CO_2_ -C g^−1^ biomass C h^−1^ under drought conditions (Fig. 4).

Due to the CO_2_ flush and consistent microbial biomass C in the first week, the qCO_2_ increased in all treatments. In drought-affected soils, only BC_aged_ addition had an effect on qCO_2_. The qCO_2_ of soils containing BC_fresh_ remained similar to the qCO_2_ of the control (1.15 and 1.14 mg CO_2_ -C g^−1^ biomass C h^−1^, respectively) after 90 days of incubation. In contrast, BC_aged_ showed a significantly higher qCO_2_ (1.76 mg CO_2_ -C g^−1^ biomass C h^−1^).

## Discussion

### Biochar effects on water conditions

We found a positive effect of BC on the water holding capacity, which was more pronounced in the BC_aged_ than in the BC_fresh_ amended soil (Fig. 2). BC_aged_ treatments showed at the end of the incubation experiment an increased volumetric water content under drought conditions compared to the other treatments. This additional water can be assigned to plant available water because the unavailable water (>pF 4.2) was not affected by BC addition (Fig. 2). We assume that fragmentation and mechanical stresses of freeze-thaw cycles during field exposure form new cracks and fractures and thus increase the pore connectivity of BC_aged_ compared to BC_fresh_ particles^22^. The additionally retained plant available water could reduce water stress and hence retard drought effects to plants^44^.

### Biochar effects on microbial activity

As found in previous studies e.g.^8,13^, total mineralization decreased with decreasing water contents in all treatments (Fig. 3a). Nevertheless, SOC mineralization decreased less in BC containing soils and was strongly dependent on the nature of BC (fresh or aged; Fig. 3a). In agreement with the literature, under optimum water conditions, we found a flush of SOC mineralization at the beginning of the experiment for treatments with BC_fresh_ (pF 1.8). This flush was observed in many studies after addition of fresh BC to soil^45–48^, and may be related to mineralization of labile C compounds of the BC and stimulation of native SOC mineralization^49^, e.g.^50^. It may indicate an immediate adaption of microorganisms to BC_fresh_ usage^51^ but is most likely a short-term effect on SOM e.g.^52^. Responsible for this fast response might be so-called ‘r-strategist’ microbes, which are adapted to respond quickly to newly available C sources, which may be present in BC in form of volatile organic matter^53,54^. These organisms re-mineralize soil nutrients and co-metabolize more refractory OM in the process (Kuzyakov *et al*., 2000; Kuzyakov, 2010).

The absence of a mineralization flush in the beginning of the experiment for BC_aged_ treatments may be explained by depletion of labile C compounds leaving behind a recalcitrant BC residue^4,49,55^. After degradation of labile BC components, it was even found that BC lowered native SOC mineralization below the level of the control samples, likely by toxic compounds of the BC or mineral adsorptive protection^56,57^. Higher native C mineralization in treatments with BC_aged_ as compared to BC_fresh_ may be explained by sorption of potential inhibitors. Extracellular reactions could consequently increase the breakdown of native SOM^58^. Increased drought intensified the effect of BC aging on native SOC mineralization (Fig. 3b).

### Microbial biomass growth and incorporation of Biochar-C

Microorganisms benefit from improved water supply during droughts due to the presence of BC as indicated by increased microbial biomass and microbial activity as compared to the control (Tables 2 and 3). The values for incorporation of BC-C into microbial biomass presented here reflect the maximum possible values as chloroform can contribute to a dissolution of BC and extraction with K_2_SO_4_ may overestimate BC-C incorporation into microbial biomass^52,59^. For all amendments, we found a general trend of decreasing microbial biomass at pF 1.8 (22% in BC_fresh_ and 38% in the control), whereas it remained rather constant at pF 3.5 (−7% in BC_fresh_ and 9% in the control) during the 90 days of incubation (Table 3). The dynamics of microbial biomass at pF 1.8 may be related to a fast consumption of C, followed by a depletion of easy available substrate. In contrast, at pF 3.5 the development of microbial biomass suffers from water stress as indicated by stagnating values.

We found no significant differences in microbial biomass between the three treatments under optimal water conditions (Table 3). However, the BC_fresh_ addition affected the dynamics of microbial biomass during the experiment, maintaining higher total biomass amounts as compared to soils containing BC_aged_ or control soils. Zhou, *et al*.^26^ found in BC amended soils an overall moderate increase in microbial biomass by 26%, which tended to decrease with increasing duration of the experiment. The increase in microbial biomass may be explained by microbial use of the labile or extractable carbon pool^60^ of the BC, and the decreasing effect with time to its exhaustion^52,61^. The porous structure of BC can be a suitable habitat for microbes (Lehmann *et al*., 2014), offering favorable microsites and protection from predators^62^. This aspect, however, tend to play a minor role, as microsites and therefore microbial biomass should increase with aging. The BC_aged_, however, showed no significant effects on microbial biomass compared to the control.

The effect of BC properties are underlined by Ameloot, *et al*.^63^ and Durenkamp, *et al*.^64^, who found even lower microbial biomass in BC amended soils than in the control and related this to BC production conditions or/and feedstocks. Wood derived BC, as used in their studies may be unfavorable for microbial colonization^56,65^.

Under drought conditions, we observed stable microbial biomass in the control soil during the 90 days incubation period. In BC_aged_ soils, microbial biomass tended to increase only during the first 28 days, whereas BC_fresh_ addition augmented microbial biomass by 37% compared to the control. These observations may be explained by more favorable water conditions in BC containing soils, combined with easily decomposable compounds in the case of BC_fresh_ addition.

In contrast to the significant BC-C mineralization (Table 2, Fig. 3a), the incorporation into microbial biomass is rather low (Table 3). Incorporation of BC-C into microbial biomass strongly varies between fresh and aged BC. Incorporation of BC_fresh_ into the microbial biomass (Table 3) demonstrates that labile BC-C was utilized not only as energy source but also as a C substrate by microorganisms^52,56,66^. Microorganisms tended to incorporate BC_fresh_ at pF 1.8, at similar amounts as observed by other authors (e.g. Kuzyakov, *et al*.^52^ (1.5 to 2.6%). In contrast, BC_aged_ seems to be preferentially mineralized, as the ^13^C content of the microbial biomass was depleted (Table 3).

Additionally, the BC-C proportion to OC released at pF 3.5 significantly increased compared to pF 1.8 (Table 2, Fig. 3c). The low but continuous proportions of BC-C released throughout the experiment in BC_aged_ treatments (Fig. 3c) indicate a persistent microbial use of BC-C. In contrast, Ameloot, *et al*.^63^ found that wood derived BC was not used as substrate for microorganisms after 1 to 4 years of field exposure.

### Effects of Biochar on metabolic efficiency

The qCO_2_ or the specific respiration rate is used to evaluate the metabolic efficiency of the soil microbial biomass. In this experiment the measured qCO_2_-values of 1.14–4.99 mg CO_2_ -C g^−1^ biomass C h^−1^ are in a comparable range to other disturbed arable soils^67^. We found the highest qCO_2_-values of 3.56–4.64 mg CO_2_ -C g^−1^ biomass C h^−1^ (pF 1.8) and 2.24–3.08 mg CO_2_ -C g^−1^ biomass C h^−1^ (pF 3.5) at the beginning of the experiment. Thereafter, the values decreased slightly in all treatments until the end of the incubation (Fig. 4). Only at day 7 of incubation, the qCO_2_ increased by 6–39% (pF 1.8) and 9–19% (pF 3.5) due to the CO_2_ flush. Decreasing qCO_2_ in our experiment can be explained by relatively constant mineralization (per h) and decreasing microbial biomass (Fig. 3a and Table 3) in contrast to many other studies, where respiration remained constant but microbial biomass increased^26^. High qCO_2_ values imply relatively ‘large’ C losses (through respiration) and less C converted to biomass, ultimately reducing the potential for long-term C sequestration in organo-mineral complexes^68,69^. However, as changes in mineralization were not in parallel to decreases in microbial biomass, this could suggest microbial community shifts. We cannot exclude changes in microbial community composition, which additionally would affect the qCO_2_ because distinct microbial groups are able to decompose and assimilate C compounds at different rates depending on their composition^70^. These have been evidenced with some taxa-specific community changes in the works by Farrell, *et al*.^53,71^,Chen, *et al*.^72^ and Gomez, *et al*.^73^. Gomez, *et al*.^73^ additionally found that as BC amendments became larger, the decrease of microbial biomass with time was alleviated. This suggests that the BC confers buffering on the microbial community. Another explanation for microbial biomass decrease at constant mineralization could be recycling of the dead microbial biomass as a labile C source. The high qCO_2_ of all treatments at pF 1.8 could further indicate nutrient gain by overflow respiration or C excretion (‘luxury consumption’, ‘waste metabolism’^74^, ‘N-mining’)^75^. This was also found for nutrient-limited conditions across a wide range of soil and litter types e.g. for N^75^.

Whereas all treatments at pF 1.8 show similar qCO_2_-values, water stress induced significant BC effects. At pF 3.5, a low qCO_2_ of 1.14 mg CO_2_ -C g^−1^ biomass C h^−1^ and 1.15 mg CO_2_ -C g^−1^ biomass C h^−1^ in control and BC_fresh_ samples could be ascribed to shifts from growth to maintenance respiration or preparation for dormancy stages as water stress is an important constraint for microorganisms. This is explained by limited substrate supply due to slow diffusion rates along the increasingly tortuous paths of thin water films or a change in the physiology of microbes as they adjust to more desiccating conditions^10^. For example, intracellular solutes are accumulated, which affect microbial growth biochemically because of high costs for osmoregulation^76^. Highly stressed microbes will then use substrate for maintenance energy requirements and not for growth^11^.

In contrast, the qCO_2_-values in soils containing BC_aged_ differ from the other two treatments, showing a qCO_2_-value of 1.76 mg CO_2_ -C g^−1^ biomass C h^−1^ under drought conditions. In general, higher qCO_2_-values suggest improved biophysical conditions for microbial activity. We assume that control soil and soil containing BC_fresh_ were strongly affected by water stress as reflected by stagnating microbial biomass and lower C-mineralization than in BC_aged_ samples. This suggests that higher available water in the latter treatment might have led to improved conditions and microbes remaining longer metabolically active with retardation of their dormant stage.

## Conclusion

We investigated water content, microbial biomass and activity under contrasting water conditions in temperate grassland soils containing similar amounts of fresh and aged BC produced by gasification. We conclude that aging of BC significantly increased plant available water in drought-affected soils. Both BC amendments led to considerable increases in SOC mineralization despite water stress, with BC_aged_ showing the greatest effects. Moreover, the BC_fresh_-treatment maintained microbial biomass, whereas the BC_aged_ treatment showed significantly increased qCO_2_ values. While representing only a small fraction of the C mineralized, BC seems to be a constantly available C source. We thus conclude that BC addition to soil is beneficial for microbial biomass and activity under drought and that these effects are increasing with time after field exposure. Our results have further implications. Particularly, the results presented in this study support the assumption that BC amendment may be a viable means of mitigating current and future water shortages in drought-affected soils under climate change, with major positive effects for available water for plant growth and microbial activity.
